# Secreted miR-34a in astrocytic shedding vesicles enhanced the vulnerability of dopaminergic neurons to neurotoxins by targeting Bcl-2

**DOI:** 10.1007/s13238-015-0168-y

**Published:** 2015-06-20

**Authors:** Susu Mao, Qi Sun, Hui Xiao, Chenyu Zhang, Liang Li

**Affiliations:** State Key Laboratory of Pharmaceutical Biotechnology, Nanjing Advanced Institute for Life Sciences (NAILS), Nanjing University School of Life Sciences, Nanjing, 210093 China; Jiangsu Engineering Research Center for microRNA Biology and Biotechnology, Nanjing, 210093 China; Center of Kidney Disease, 2nd Affiliated Hospital, Nanjing Medical University, Nanjing, 210003 China

**Keywords:** astrocyte, shedding vesicles, miR-34a, dopaminergic neurons, Bcl-2

## Abstract

MicroRNAs (miRNAs) are a class of noncoding RNAs that regulates target gene expression at posttranscriptional level, leading to further biological functions. We have demonstrated that microvesicles (MVs) can deliver miRNAs into target cells as a novel way of intercellular communication. It is reported that in central nervous system, glial cells release MVs, which modulate neuronal function in normal condition. To elucidate the potential role of glial MVs in disease, we evaluated the effects of secreted astrocytic MVs on stress condition. Our results demonstrated that after Lipopolysaccharide (LPS) stimulation, astrocytes released shedding vesicles (SVs) that enhanced vulnerability of dopaminergic neurons to neurotoxin. Further investigation showed that increased astrocytic miR-34a in SVs was involved in this progress via targeting anti-apoptotic protein Bcl-2 in dopaminergic neurons. We also found that inhibition of astrocytic miR-34a after LPS stimulation can postpone dopaminergic neuron loss under neurotoxin stress. These data revealed a novel mechanism underlying astrocyte-neuron interaction in disease.

**Electronic supplementary material:**

The online version of this article (doi:10.1007/s13238-015-0168-y) contains supplementary material, which is available to authorized users.

## INTRODUCTION

Parkinson’s disease (PD) is characterized by the progressive degeneration of dopamine (DA) neurons in the substantia nigra (SN). Circumstantial evidence has shown that the neuron-glia interaction plays an important role in PD pathogenesis (Furman and Norris, 2014; Lee et al., 2013; Oeckl et al., 2012). It has been suggested that reactive astrocytes may amplify the devastating effects of PD by accelerating the dopaminergic (DA) neuron loss (Saijo et al., 2009).

Microvesicles (MVs) were originally described as cellular debris with limited biological function (Morel et al., 2004). However, increasing amounts of evidence suggest that MV shedding is a constitutive mode of intercellular communication (Al-Nedawi et al., 2009; Gupta and Pulliam, 2014; Hu et al., 2012; Keller et al., 2006; Shantsila et al., 2010). MVs can be divided into two groups—shedding vesicles (SVs) and exosomes—with different discharging processes (Bianco et al., 2009). The release of shedding vesicles is a result of direct budding from the plasma membrane, while exosomes are produced by exocytosis of multivesicular bodies (Zomer et al., 2010). Bianco F et al. showed that in the central nervous system, glial cells can release both type of MVs (Bianco et al., 2009). Further investigation produced evidence that MVs released from microglia stimulate synaptic activity via enhanced sphingolipid metabolism, indicating that microglia MVs may have significant biological functions under normal conditions (Antonucci et al., 2012). Our previous work showed that exosomes can deliver microRNAs (miRNAs) into endothelial cells and then modulate the migration of the cell by repressing the target protein expression (Zhang et al., 2010). In recent years, accumulating evidence also showed that secreted miRNAs in MVs have significant biological functions including effects on cell proliferation, development, differentiation as well as cell death and cancer progression (Hu et al., 2012; Janowska-Wieczorek et al., 2005; Jung and Suh, 2014; Liu et al., 2013; Vlassov et al., 2012; Zhou et al., 2014; Zhu and Fan, 2011). However, there are few studies regarding the function of secreted miRNAs in neuron-glia interaction of the central nervous system either in physiological or disease conditions. Whether secreted miRNAs in astrocytic MVs are involved in PD pathogenesis is still unknown; therefore it is important to investigate the potential role of MVs in astrocyte-neuron interactions in PD.

In the present study, we used both *in vitro* and *in vivo* PD models to evaluate the effects of secreted astrocytic MVs under different conditions on DA neurons survival. We demonstrated that astrocytes under conditions of LPS stress released SVs, which enhanced the vulnerability of DA neurons to neurotoxin. We also found evidence that miR-34a was increased in astrocytic SVs after LPS stimulation and that miR-34a then entered DA cells and repressed the anti-apoptotic protein Bcl-2 (Hockenbery et al., 1993; Wang et al., 2009; Yang et al., 1997; Zhou et al., 2014), thus compromising cellular resistance to neurotoxins. In addition, we showed that blocking the astrocytic miR-34a can rescue the anti-apoptotic function of DA neurons *in vitro* and alleviate DA neuron loss as well as abnormal behavior induced by apomorphine under 6-OHDA stress *in vivo*. These data suggest a new mechanism by which astrocytes influence neuronal survival under disease conditions.

## RESULTS

### Biochemical and morphological characterization of distinct types of microvesicles released from astrocytic cells

**Figure 1:**
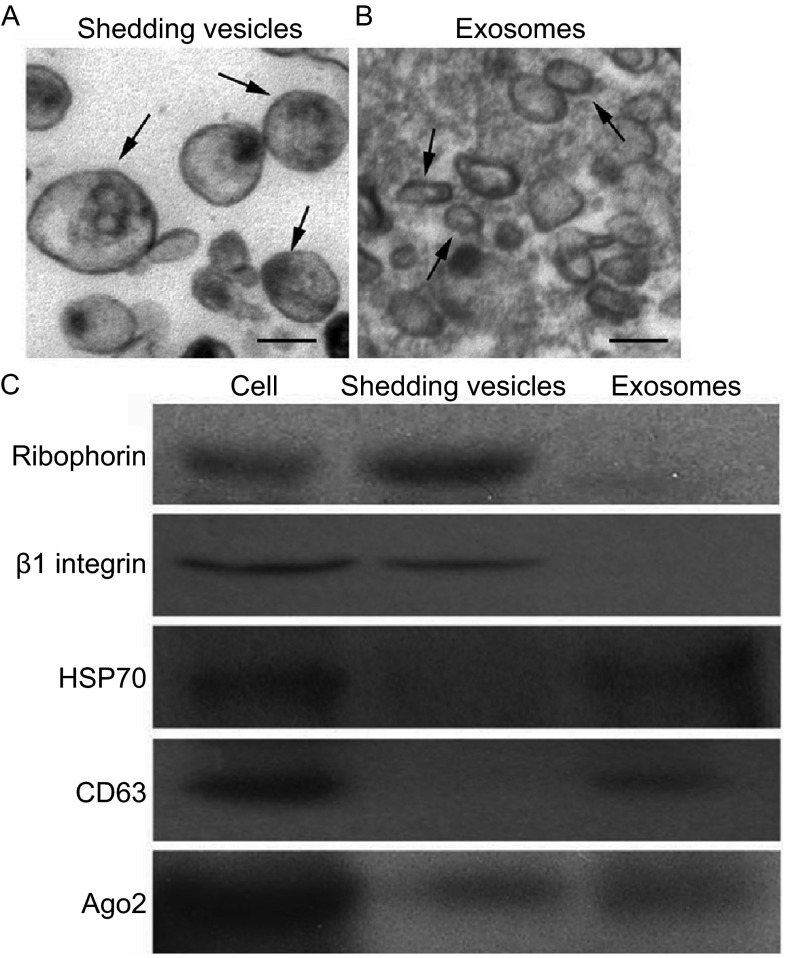
**Morphology and characterization of purified microvesicles of U-87 MG cells**. (A) EM imaging of purified shedding vesicles secreted by U-87 MG cells (arrows, scale bar = 100 nm); (B) EM imaging of purified exosomes secreted by U-87 MG cells (arrows, scale bar = 100 nm); (C) Western blot of the molecular markers of shedding vesicles (Ribophorin and β1 integrin) and exosomes (CD63 and HSP70); both types of microvesicles contained the miRNA-associated protein Ago2

We collected the shedding vesicles (SVs) and exosomes from the culture medium by differential ultracentrifugation. EM imaging showed the different sizes of the distinct types of microvesicles. SVs consisted of cup-shaped vesicles in the range of 100–200 nm, while exosomes contained relatively small vesicles of approximately 30–80 nm (Fig. 1A and 1B).

We also analyzed the different types of vesicles by Western blotting of some SVs and exosome markers. Typical SV markers, such as β1 integrin and ribophorin, were enriched in our 10,000 ×*g* pellet, while exosome markers, such as CD63 and heat shock protein HSP70, were found mainly in the 110,000 ×*g* pellet. Furthermore, we also found Ago2 protein expression in both types of the releasing microvesicles (Fig. 1C). Ago2 is reported to be associated with functional miRNAs, indicating that the released microvesicles might have carried miRNAs with Ago2 proteins.

### Astrocyte-derived shedding vesicles under stress conditions enhanced the vulnerability of DA neurons to neurotoxins

**Figure 2:**
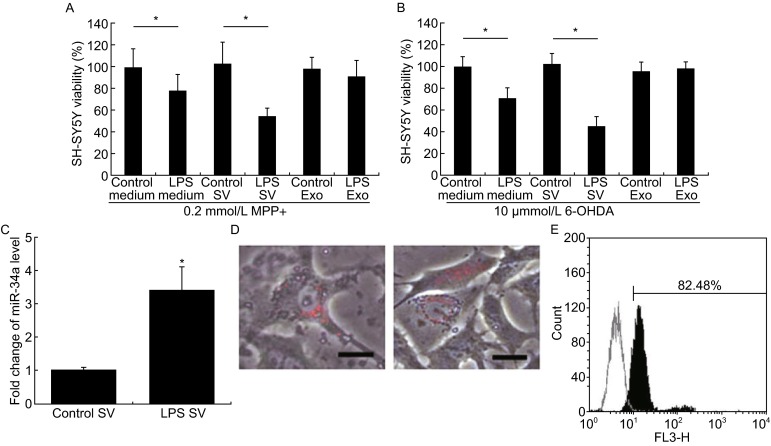
**SVs derived from LPS-stimulated U-87 MG cells contain increased miR-34a and reduce SH-SY5Y cell viability to neurotoxins**. (A) Cell viability assessed by CCK-8 showed that pretreatment with LPS SVs increased the vulnerability of SH-SY5Y cells to 0.2 mmol/L MPP+ treatment, *P < 0.05; (B) Cell viability assessed by CCK-8 showed that pretreatment with LPS SVs increased the vulnerability of SH-SY5Y cells to 10 μmol/L 6-OHDA treatment, *P < 0.05. Control SV: SVs derived from PBS-stimulated U-87 MG cells; LPS SVs: SVs derived from LPS-stimulated U-87 MG cells; Control Exo: Exosomes derived from control U-87 MG cells; LPS Exo: Exosomes derived from LPS-stimulated U-87 MG cells; (C) Relative miRNA levels between control SV and LPS SVs, *P < 0.05; (D) Fluorescent images showed that labeled SVs derived from U-87 MG cells can enter SH-SY5Y cells after co-incubation; red dots are labeled shedding vesicles, Scale bar = 10 μm; (E) FACS analysis showed that more than 80% of SH-SY5Y cells carry red fluorescence after co-incubation with labeled SVs; the left histogram represents the control group; the right histogram represents SH-SY5Y cells co-cultured with the fluorescence-labeled SVs

Next, we investigated the impact of astrocyte-derived microvesicles under stress conditions on cell survival. We found that neither SVs nor exosomes derived from the LPS-stimulated U87-MG astroglial cell line had any effects on SH-SY5Y cell viability under normal conditions (Fig. S1). However, further investigation showed that pretreatment of SVs derived from LPS-stimulated U87-MG cells increased the vulnerability of the SH-SY5Y cells to threshold concentrations of neurotoxins, such as 0.2 mmol/L MPP+ or 10 μmol/L 6-OHDA (Figs. 2A, 2B and S1).

### MiRNA profiling of SVs derived from LPS-stimulated U-87 MG cells

We collected the SVs from LPS (LPS SVs) and PBS (control SV) treated U-87 MG cells, respectively. In the present study, we first analyzed the miRNA levels in LPS SV and control SV using miRNA array. We focused on those miRNAs with a fold-change (LPS SV/control SV) >2 or <0.5, respectively. In each group, 10 miRNAs were listed by their signal intensity in a descending manner (Table S1). Among these altered miRNAs profiles, miR-34a was of particular interest, as it was upregulated 2.86-fold in LPS SV, and more so, as one of its predicted genes, Bcl-2, has been demonstrated to regulate cell apoptosis (Hockenbery et al., 1993; Yang et al., 1997). We further assessed the expression level of miR-34a in LPS SV by qPCR. Consistent with the result of miRNA array, we found that miR-34a was significantly increased, with a fold change of 3.4 ± 0.7 (Fig. 2C). Because it has been reported that miR-34a targets Bcl-2 protein, which regulates cell apoptosis, we proposed that up-regulation of miR-34a in LPS SVs may contribute to the increased vulnerability of SH-SY5Y cells to neurotoxins via repressing Bcl-2 expression.

### Secreted miR-34a in LPS SVs enhanced the vulnerability of SH-SY5Y cells to neurotoxins by repressing Bcl-2 protein expression

We co-cultured the fluorescence-labeled SVs of U-87 MG cells with SH-SY5Y cells and found labeled SVs in cultured SH-SY5Y cells by fluorescence microscopy (Fig. 2D). FACS analysis also showed that the 82.48% of cultured SH-SY5Y cells were positive for fluorescence-labeled SVs (Fig. 2E). These results indicated the possibility that SVs may be able to deliver miRNAs into target cells by co-culture.

**Figure 3:**
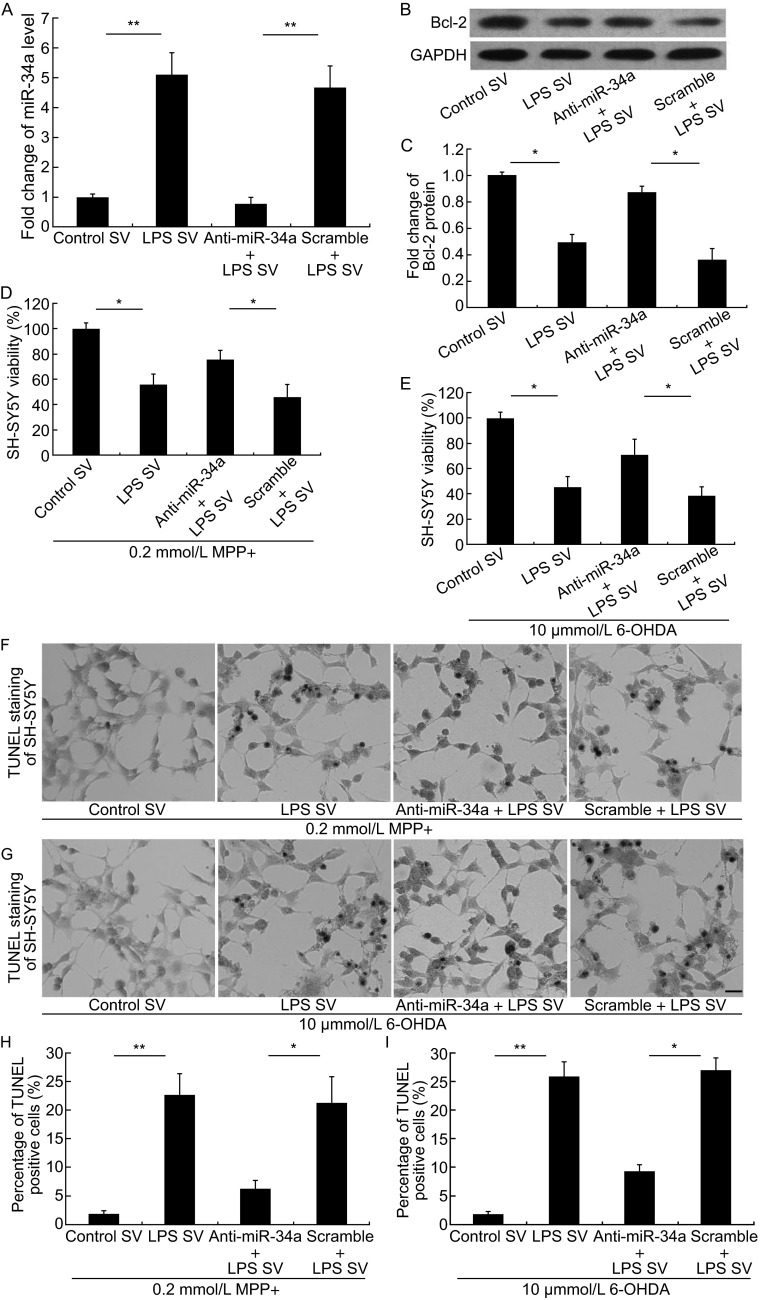
**Co-incubation with LPS SVs increases miR-34a level while down-regulates Bcl-2 level that further compromises neurotoxin resistance of SH-SY5Y cells**. (A) QPCR analysis of miR-34a levels in SH-SY5Y cells after co-incubation with different groups of SVs, **P < 0.01; (B and C) Western-blot and quantification of Bcl-2 protein levels in SH-SY5Y cells after co-incubation with different groups of SVs, *P < 0.05; (D and E) Viability of SH-SY5Y cells pre-treated with different groups of SVs following 0.2 mmol/L MPP+ or 10 μmol/L 6-OHDA stress, *P < 0.05; (F and G) TUNEL staining of SH-SY5Y cells pre-treated with different groups of SVs after 0.2 mmol/L MPP+ or 10 μmol/L 6-OHDA stress, scale bar = 50 μm; (H and I) Percentage of TUNEL positive cells among SH-SY5Y cells pre-treated with different types of SVs after 0.2 mmol/L MPP+ or 10 μmol/L 6-OHDA stress, *P < 0.05, **P < 0.01; Control SV: SVs derived from control U-87 MG cells; LPS SV: SVs derived from LPS-stimulated U-87 MG cells; anti miR-34a + LPS SV: SVs derived from LPS-stimulated U-87 MG cells transfected with miR-34a inhibitor; Scramble + LPS SV: SVs derived from LPS-stimulated U-87 MG cells transfected with scramble RNA

As it has been reported that miR-34a may target Bcl-2 protein, we first performed the luciferase assay and Western blot in SH-SY5Y cells which showed that miR-34a can indeed target Bcl-2 mRNA directly and repress Bcl-2 protein levels in SH-SY5Y cells (Fig. S2). Next, we further investigated the role of secreted miR-34a in neuronal loss under neurotoxin treatment. We found that the level of mature miR-34a was significantly increased in SH-SY5Y cells after the treatment with LPS SVs (Fig. 3A). As a result, the protein level of Bcl-2 was repressed in these cells (Fig. 3B and 3C). However, pretreatment with anti-miR-34a in U-87 MG cells blocked the increase of miR-34a and negated the repression of Bcl-2 caused by LPS SVs (Fig. 3A–C). Further investigation showed that cellular viability was reduced and apoptotic ratio was increased in LPS SV-treated SH-SY5Y cells after 0.2 mmol/L MPP+ exposure. On the other hand, pretreating U-87 MG cells with anti-miR-34a significantly lessened the effect of LPS SVs on SH-SY5Y cell viability and apoptotic ratio (Fig. 3D, 3F and 3H). Similar results were achieved when 6-OHDA was used (Fig. 3E, 3G and 3I).

**Figure 4:**
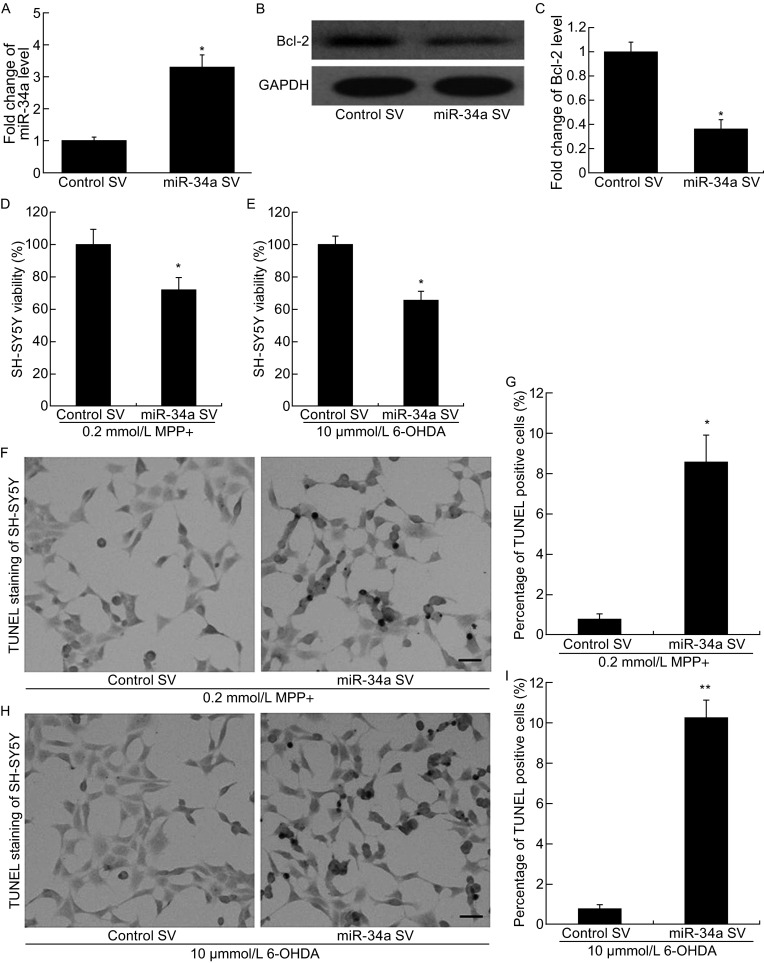
**Overexpression of miR-34a in SVs is sufficient to increase the vulnerability of SH-SY5Y cells to neurotoxins**. (A) QPCR analysis of miR-34a levels in SH-SY5Y cells after co-incubation with miR-34a SV compared with cells co-incubated with control SV, *P < 0.05; (B and C) Western-blot and quantification of Bcl-2 protein levels in SH-SY5Y cells after co-incubation with miR-34a SV compared with cells co-incubated with control SV, *P < 0.05; (D and E) Viability of SH-SY5Y cells pre-treated with different groups of SVs after 0.2 mmol/L MPP+ or 10 μmol/L 6-OHDA stress, *P < 0.05; (F and H) TUNEL staining of SH-SY5Y cells pre-treated with different groups of SVs after 0.2 mmol/L MPP+ or 10 μmol/L 6-OHDA stress, scale bar = 50 μm; (G and I) Percentage of TUNEL positive cells of SH-SY5Y cells pre-treated with different types of SVs after 0.2 mmol/L MPP+ or 10 μmol/L 6-OHDA stress, *P < 0.05, **P < 0.01. Control SV: SVs derived from control U-87 MG cells; miR-34a SV: SVs derived from U-87 MG cells that overexpressed miR-34a

In addition, we transfected miR-34a into U-87 MG cells and then collected the SVs (miR-34a SVs) without LPS stimulation. In these SVs, miR-34a level was increased by approximately 22.1 ± 2.7 fold (Fig. S3). Pretreatment with miR-34a SVs increased the levels of mature miR-34a in SH-SY5Y cells, leading to a decrease in Bcl-2 protein levels (Fig. 4A–C). Moreover, we also observed that miR-34a SVs reduced the cellular viability and increased the percentage of apoptotic SH-SY5Y cells after 0.2 mmol/L MPP+ or 10 μmol/L 6-OHDA treatment (Fig. 4D–I).

Together, these data provide strong evidence that secreted miR-34a in LPS SVs plays a major role in regulating apoptosis of SH-SY5Y cells exposed to neurotoxins by repressing Bcl-2 protein expression.

### SVs derived from LPS-stimulated astrocytes increased primary DA neuron loss after neurotoxin exposure

**Figure 5:**
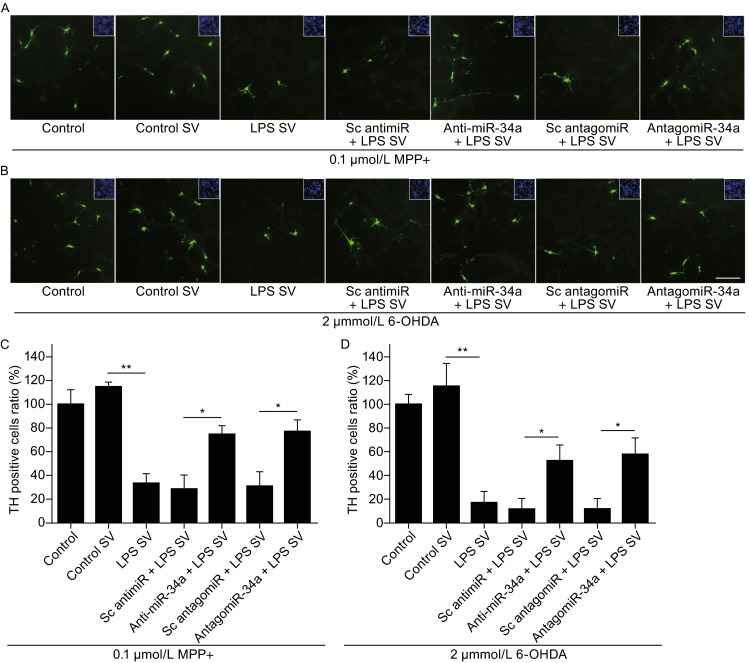
**SVs derived from LPS-stimulated primary astrocytes enhance DA neurons loss after treatment with neurotoxins**. (A and C) TH staining and quantification of the percentage of TH-positive cells among primary neurons pre-treated with different groups of SVs after 0.1 μmol/L MPP+ stress, *P < 0.05, **P < 0.01. Inset: DAPI staining of the cell nuclei in the field of view. (B and D) TH staining and quantification of the percentage of TH-positive cells among primary neurons pre-treated with different groups of SVs after 2 μmol/L 6-OHDA stress, *P < 0.05, **P < 0.01, scale bar = 200 μm. Inset: DAPI staining of the cell nuclei in the field of view. Control: a control group of neuron culture without SVs incubation; Control SV: SVs derived from primary astrocytes; LPS SV: SVs derived from LPS-stimulated primary astrocytes; anti-miR-34a + LPS SV: SVs derived from LPS-stimulated primary astrocytes transfected with miR-34a inhibitor; Sc antimiR + LPS SV: SVs derived from LPS-stimulated primary astrocytes transfected with scramble RNA; Sc antagomiR + LPS SV: SVs derived from LPS-stimulated primary astrocytes, and the primary neurons were pretreated with scramble antagomiR; antagomiR-34a + LPS SV: SVs derived from LPS-stimulated primary astrocytes, and the primary neurons were pretreated with antagomiR-34a

Here, we further used primary culture to verify the results demonstrated in the cell lines. We collected LPS SVs from primary astrocytes and found that the miR-34a levels were also increased (Fig. S4). Pretreatment with primary astrocytic LPS SVs caused significant TH-positive cell loss after low concentration MPP+ (0.1 μmol/L) or 6-OHDA (2 μmol/L) treatment, without significant effect on the total neuron number showed by DAPI (Fig. 5A–D). This effect was strongly attenuated after blocking the up-regulation of miR-34a in astrocytic LPS SVs by anti-miR-34a pretreatment in astrocytes. To further verify that miR-34a is necessary and sufficient to induce apoptosis, we also used antagomiR oligonucleotides directed against miR-34a (antagomiR-34a) to block the level of miR-34a in primary neurons. Similar results were obtained (Fig. 5A–D). These results indicated that in an *ex vivo* situation, stressed astrocytes released SVs that increased TH neuron vulnerability, in which an increased level of secreted miR-34a may be involved.

### Secreted miR-34a in LPS SVs enhanced the DA neuron vulnerability to 6-OHDA *in vivo*

**Figure 6:**
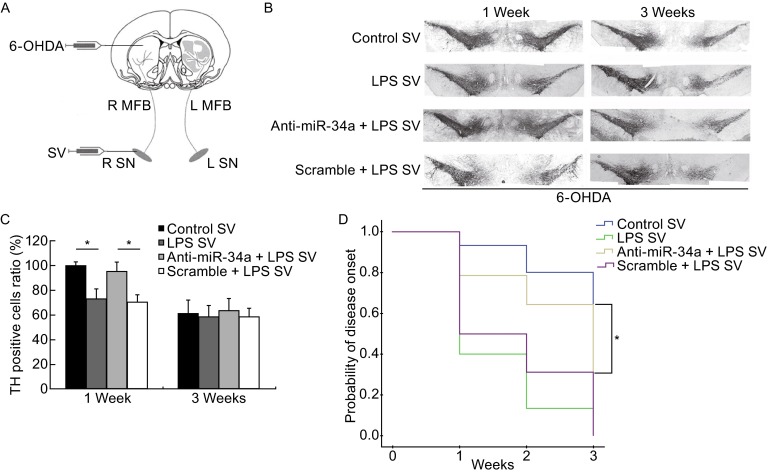
**SVs derived from LPS-stimulated primary astrocytes accelerates DA neurons loss as well as disease onset in 6-OHDA rat model**. (A) Schematic diagram of the experiment process; (B) TH-immunoreactive cell bodies in SN of different groups of rats; (C) Quantification of DA neurons loss among different groups of rats, *P < 0.05 (*n* = 10 in each group); (D) The Kaplan-Meier curves demonstrate the probability of disease onset, *P < 0.05. (*n* = 15 in each group)

To further understand the role of LPS SVs *in vivo*, we evaluated the effects of different groups of SVs in the 6-OHDA rat model. We injected 6-OHDA unilaterally into the striatum while simultaneously injecting SVs into the SN in the same side (Fig. 6A). We found that the number of DA neurons was significantly decreased in the LPS SVs-treated rats after 1 week, while blocking the up-regulation of miR-34a in LPS SVs can reduce the DA neuron loss at the same time point. However, when we examined DA neurons 3 weeks after the injection, we found that all groups of rats showed severe DA neuron loss with no significant differences (Figs. 6B and 2C). We further assessed apomorphine-induced contralateral rotation of these rat groups at different time points (1 week, 2 weeks and 3 weeks respectively) after the surgery. Among 15 rats for each group that exhibit abnormal apomorphine-induced rotation at the third week (>6/min), we found that the probability of disease onset of the anti-miR-34a + LPS SV group is 3/15 on the first week and 5/15 on the second week while the numbers in scramble + LPS SV group are 8/15 and 11/15 respectively (Fig. 6D). These results showed that blocking the up-regulation of miR-34a in LPS SVs can significantly delay the disease onset but not able to reverse the disease progression after the injection.

Taken together, our data suggest that LPS-stimulated astrocyte SVs can enhance DA neuron vulnerability, which accelerates the progression of DA neuron loss, and that miR-34a plays a crucial role in this process.

## DISCUSSION

Neuron-glia interaction is thought to play an important role in the pathogenesis of many neurodegenerative diseases (Malavolta et al., 2013; Saijo et al., 2009). In the present study, we revealed a novel mechanism by which astrocytes can influence neuronal survival under PD conditions. Our results demonstrated that reactive astrocytes release SVs (not exosomes), which increase the percentage of apoptotic SH-SY5Y cells after treatment with low concentrations of neurotoxins. Thus, these SVs may increase DA neuron vulnerability. Furthermore, we demonstrated that there are differences in miRNA expression patterns between SVs produced by reactive and resting astrocytes and that miR-34a was significantly up-regulated. Our previous work demonstrated that MVs can deliver miRNAs into target cells and shut down target gene expression (Zhang et al., 2010). In addition, several studies revealed that MVs mediated cell interaction exists in central nervous system (Antonucci et al., 2012; Hu et al., 2012). Moreover, there is evidence that miR-34a targets the anti-apoptotic protein, Bcl-2, which can further induce tubular cell apoptosis (Liu et al., 2011; Sun et al., 2012; Wang et al., 2009; Zhou et al., 2014). Here we also performed luciferase assay which ensure that miR-34a can directly bind and regulate Bcl-2 in SH-SY5Y cells. These data together indicate the possibility that astrocytic miR-34a may be transferred into neurons via SVs, where it then represses Bcl-2 protein levels and decreases the anti-apoptotic function of the targeted neurons. Notably, several other miRNAs listed in Table S1 also have important functions in modulating cell apoptosis. Among up-regulated miRNAs, miR-18a was validated to regulate cell apoptosis by targeting Bcl-2 directly in mice and humans (Scherr et al., 2014), and miR-296-3p was found to increase apoptosis levels of an islet cell line αTC1-6 cells (Barbagallo et al., 2013). On the other hand, in those down-regulated miRNAs, inhibition of miR-708 promoted the apoptosis of SW480 cells (Lei et al., 2014) and T24 cells (Song et al., 2013). These data suggest that in addition to miR-34a, those altered miRNAs summarized in Table S1 may also affect cell apoptosis. Nevertheless, our data demonstrated that the level and variation of miR-34a in astrocytic SVs is one of the highest among these miRNAs upon LPS stimulation, which indicates that miR-34a plays a major role in modulating cell apoptosis.

Furthermore, we provided direct evidence that fluorescence-marked astrocytic SVs can enter target cells after 24 h of incubation, increase miR-34a levels and decrease Bcl-2 protein levels in SH-SY5Y cells. Blocking the increase in the miR-34a levels in astrocytic SVs by a miR-34a inhibitor alleviated the DA cell loss caused by LPS-stressed astrocytic SVs. This evidence indicates that increased miR-34a levels may contribute to the disruptive effect of reactive-astrocytic SVs on DA cell survival. Since our miRNAs array data indicated that the level of several other miRNAs also changed in astrocytic SVs after LPS stimulation, we further testify the effect of miR-34a by over-expression experiment. When we increased the miR-34a levels in astrocytic SVs without LPS stimulation by transfecting astrocytes with pre-miR-34a, we found that those SVs still enhanced the vulnerability of DA neurons to low concentrations of neurotoxins, leading to an increased percentage of apoptotic cells. These results strongly indicated that the upregulation of miR-34a in astrocytic SVs is sufficient to increase neuronal vulnerability under disease conditions.

In primary culture, we found that LPS-stimulated rat astrocytes also generated SVs with higher levels of miR-34a. In addition, SVs derived from LPS-stressed primary astrocytes enhanced primary DA neuron vulnerability to low concentrations of both MPP+ and 6-OHDA. This disruptive effect was alleviated when we blocked the increase of miR-34a using a miR-34a inhibitor. All of these results were consistent with the data obtained from the cell lines, which suggests that a similar mechanism exists for both primary and cell line culture systems.

Next, we investigated the role of secreted miR-34a in astrocytic SVs in regulating neuron survival *in vivo*. Using the 6-OHDA model, we demonstrated that SVs derived from LPS-stimulated primary astrocytes significantly accelerated DA neuron loss following 6-OHDA treatment. Most of the rats lost more than 30% of their DA neurons in the first week and showed apomorphine-induced rotation. When we blocked the increase of miR-34a in astrocytic SVs by transfecting miR-34a inhibitor into primary astrocytes, we found that the acceleration of DA neuron loss was reduced and that apomorphine-induced rotation was delayed compared with scramble group. These data indicate that the increased levels of secreted miR-34a in LPS-stimulated-astrocytic SVs play a crucial role in the acceleration of DA neuron loss. Recently, it is reported that miR-34a is shuttled in exosomes which induces cell apoptosis in fibrotic kidney (Zhou et al., 2014). This is similar with our findings in nervous system except that in our model we demonstrated that SVs contain the increase of miR-34a and display robust effect rather than exosomes in regulating cell apoptosis. When we examined those PD rats three weeks after 6-OHDA injection, we found all groups showed severe DA neuron loss. Combined with behavioral tests, it is indicated that blocking the function of secreted miR-34a in LPS-stimulated astrocytic SVs can postpone but not reverse the progress of disease.

In many but not all cases of Parkinson’s disease, an increase in the number of reactive astrocytes has been observed (Forno et al., 1992), which suggest that astrocytes play vital roles in PD. In the 6-OHDA animal model of PD, Stromberg found an increased amount of reactive astrocytes in the striatum (Stromberg et al., 1986). Activation of astrocytes is a prevalent response to neuronal damage in neurodegenerative diseases, with potential neurotoxic and neuroprotective consequences. Several findings suggest a neuroprotective role for astrocytes in PD as well as animal models of PD. Saura et al. found infusion of IL-1β led to an increased activation of astrocytes in the the substantia nigra and the dopaminergic neurons were protected against 6-OHDA (Saura et al., 2003). It also has been shown that astrocytes can protect neurons from NO toxicity by a glutathione dependent mechanism (Chen et al., 2001). Additionally, astrocytes express monoamine oxidase-B and catechol-O-methyl-transferaseare, thus reducing the oxidative stress by metabolizing dopamine (Hirsch et al., 1999). On the other hand, Li et al. found that suitable activation of astrocytes increased their protection effect on dopaminergic neurons stimulated by LPS, while excessive activation attenuated it, which suggested that astrocytes played a double-faced effect on dopaminergic neurons (Li et al., 2009). Also, astrocytes can release chemokines and cytokines that are deleterious to neurons in various models of PD, such as reactive oxygen species, nitrite, S100B and so on (Niranjan et al., 2010). The present study suggested that astrocytes had a destructive effect through SV-miR-34a-Bcl2 pathway under LPS stress condition. These results suggest that in stress condition, activated astrocytes may act as a double-edged sword in regulating DA neurons survival. Such functional balance of reactive astrocytes needs to be further explored. Altogether, our results suggested that under stress conditions, such as LPS stimulation, increased levels of miR-34a in astrocytic SVs can be transported into DA neurons, where they enhance the vulnerability of the neurons to neurotoxins by targeting the anti-apoptotic protein Bcl-2. The increase of astrocytic miR-34a in disease conditions may be a potential target for alleviation of PD progress.

## MATERIALS AND METHODS

### Animals and cell culture

All of the animal care and experimental procedures were performed in accordance with the Laboratory Animal Care Guidelines approved by the Model Animal Research Center of Nanjing University. U-87 MG and SH-SY5Y cell lines were maintained in Dulbecco’s Modified Eagle Medium: Nutrient Mixture F-12 (DMEM/F-12, Life Technologies, Grand Island, NY, USA) with 10% Fetal Bovine Serum (FBS, Life Technologies). Primary cultures of rat brain astrocytes were prepared as follows: briefly, newborn (P0) rat cerebral cortices were separated in Hank’s Balanced Salt Solution (HBSS, Life Technologies) and digested into cell suspensions. Digestion was terminated by the addition of 10% FBS. Cells were seeded in 75 cm^2^ flask and maintained in DMEM/F-12 medium containing 10% FBS. After 14 days of culture, the astrocytes were separated from the microglia and oligodendrocytes by shaking for 12 h in an orbital shaker at 240 rpm. Cell samples were checked regularly for the expression of glial fibrillary acid protein, a marker for astrocytes. Only those cultures containing >95% astrocytes were used. Primary cultures of rat DA neurons were prepared as follows: briefly, embryonic (E14.5) rat ventral mesencephalon (VM) was removed from the brains in HBSS and digested into cell suspensions. Digestion was terminated by the addition of 10% FBS. Cells were seeded in 24-well plates that were coated with 0.05% poly-D-lysine (Sigma-Aldrich, St Louis, MO) and maintained in neurobasal medium (Life Technologies) containing 2% B27 (Life Technologies) and 0.2 mmol/L L-glutamine (Life Technologies) for 4 more days *in vitro* before use. U-87 MG cells and primary astrocytes were treated with different concentrations of LPS (Sigma-Aldrich) for 6 h and washed three times with PBS. The conditioned medium was collected after another 48 h culture. SH-SY5Y cells and primary DA neurons were treated with different groups of astrocytic SVs in an astrocyte-to-neuron ratio of 9:1 for 24 h before exposure to neurotoxin. SH-SY5Y cells were exposed to different concentrations of 1-Methyl-4-phenylpyridinium iodide (MPP+, Sigma-Aldrich) or 6-Hydroxydopamine hydrobromide (6-OHDA, Sigma-Aldrich) for 6 h and cultured for another 24 h before subsequent assays. Primary DA neurons were exposed to different concentrations of MPP+ or 6-OHDA for 1 h and cultured for another 24 h before subsequent assays.

### MVs isolation and electronic microscopy

MVs were isolated from culture medium by differential centrifugation as in previous publications (Bianco et al., 2009). After removing cells and other debris by centrifugation at 300 ×*g* and 1200 ×*g*, the supernatant was centrifuged at 10,000 ×*g* for 1 h to collect the shedding vesicles (SVs) and at 110,000 ×*g* for 2 h to collect the exosomes; all steps were performed at 4°C. MVs were collected from the pellet and resuspended in FBS-free medium. In preparation for Electron Microscope (EM) imaging, MVs were precipitated, fixed in a 0.1 mol/L phosphate buffer containing 2.5% glutaraldehyde (Sigma-Aldrich), and cut into 50 µm-thick sections using a vibratome. The sections were postfixed with 1% OsO_4_, dehydrated and embedded in Durcupan (ACM; Fluka, Buchs, Switzerland) on a microscope slide and covered with a coverslip. The sections were cut again using a Reichert ultramicrotome into 70-nm-thick sections. The ultrathin sections were then stained with uranyl acetate and lead citrate and evaluated with an electron microscope.

### RNA isolation, microRNA array and quantitative real-time PCR of mature miRNAs

The total RNA of the MVs derived from 10^8^ cells was extracted using TRIzol Reagent (Life Technologies). Samples of total RNA were extracted for miRNAs Affymetrix GeneChip miRNA 3.0 array analysis (Life Technologies). Quantitative real-time PCR (qPCR) was performed using TaqMan microRNA probes (Applied Biosystems, Foster City, CA, USA) according to the manufacturer’s instructions. Briefly, 1 μg of the total RNA was reverse-transcribed to produce cDNA using Avian Myeloblastosis Virus reverse transcriptase (Takara, Dalian, China) and stem-loop RT primers (Applied Biosystems). Real-time PCR was performed using a TaqMan PCR kit and an Applied Biosystems 7300 Sequence Detection System (Applied Biosystems). All of the reactions, including the no-template controls, were run in triplicate. After the reactions, the CT values were determined using fixed-threshold settings.

### Fluorescence labeling of SVs

U-87 MG cells were treated with SynaptoGreen (Sigma-Aldrich) for 6 h and washed five times with PBS; after the cells were cultured for another 48 h, the medium was collected and the SVs were isolated. The collected SVs were added into the SH-SY5Y cells in an astrocyte-to-neuron ratio of 9:1 for 24 h. Subsequently, the SH-SY5Y cells were collected and analyzed by fluoromicroscopy or Fluorescence Activated Cell Sorter (FACS).

### Cell transfection with ncRNA, anti-miR-34a or pre-miR-34a

U-87 MG cells, primary astrocytes and primary neurons were transfected using Lipofectamine 2000 (Life Technologies) according to the manufacturer’s instructions. For overexpression of miR-34a, 50 nmol/L of pre-miR-34a or scrambled negative control pre-miRNA (pre-ncRNA) was used. For knockdown of miR-34a, 20 nmol/L of anti-miR-34a, 50 nmol/L of antagomiR oligonucleotides directed against miR-34a or scrambled negative control anti-miRNA (anti-ncRNA) was used. Cell media were collected 48 h after transfection.

### Luciferase assay

The 3′UTR of Bcl-2 containing the predicted target sequence was cloned and inserted into pMIR-REPORT^TM^-Luciferase vector (Ambion, Austin, TX, USA). Forward primer: 5′-CATGCCTGCCCCAAACAAATA-3′; reverse primer: 5′-AGGGCATTTTTCCCATCGCT-3′. Mutated vector was generated in Invitrogen by replacing the predicted target region with its reverse sequence (from ACUGCC to TGACGG). SH-SY5Y cells were seeded in 24 well plates for 12 h. Afterwards, 0.2 μg of firefly luciferase reporter plasmid, 0.2 μg of β-galactosidase (β-gal) expression vector (Ambion), and 50 nmol/L of miR-34a mimics or scrambled control were transfected into cells. Cells were harvested for luciferase assay (Promega, Madison, WI, USA) 24 h later, and luciferase activities were normalized to β-gal activities.

### CCK-8 Assay

Cytotoxicity was also assayed using CCK-8 (Dojindo, Kumamoto, Japan) assay. Briefly, SH-SY5Y cells were cultured in a 96-well plate with different treatments. The cells were then treated with 10 μL CCK-8 solution per well and incubated for 3 h at 37°C. The amount of formazan dye generated by cellular dehydrogenase activity was measured by absorbance at 450 nm with a microplate reader.

### TUNEL assay

For the TUNEL assay, the cells were harvested and washed with PBS, fixed and permeabilized, and then TUNEL-labeled using a One Step TUNEL Apoptosis Assay Kit (Beyotime, China) as instructed. The total percentage of apoptotic cells was estimated by determining the percentage of cells with positive TUNEL staining in five randomly selected fields from each slide under a microscope.

### TH staining

After treatment, primary DA cultures were washed with PBS and fixed with 4% paraformaldehyde (PFA) for 15 min. Then, the cells were blocked in PBS with 5% horse serum and 0.03% Triton X-100 for 1 h. Immunocytochemistry was performed using mouse anti-tyrosine hydroxylase monoclonal antibody (Sigma). The percentage of cells with positive TH staining was estimated in five randomly selected fields under a fluorescent microscope.

Experimental animals were anesthetized and perfused transcardially with 0.9% saline followed by 4% PFA. Brain samples were postfixed with 4% PFA overnight and equilibrated in 15% and 30% sucrose. Coronal sections of 40 μm were prepared with a sliding microtome. Immunohistochemistry was performed using mouse anti-tyrosine hydroxylase monoclonal antibody. Unbiased quantification of TH-immunoreactive neurons in the SN was performed according to the optical fractionator principle (Gundersen et al., 1988).

### Intracerebral injection

Animals received two 2.5-μL stereotaxic injections of 2.0 μg/μL 6-OHDA (total dose = 10 μg 6-OHDA) delivered at a rate of 0.5 μL/min and the syringe was left in place for an extra 5 min and then withdrawn gently, and the skin was sutured. Striatum injection coordinates were as follows: site 1: AP +1.3, ML −2.8, DV −4.5; site 2: AP −0.6, ML −4.0, DV −5.5; and tooth bar set at −3.2. The lesion was allowed to progress for 1–3 weeks after that animals were killed for further analyses.

The vesicles (1 μg/μL, protein concentration) were injected at two sites (site 1: AP −4.8, ML −2.0, DV −7.1; site 2: AP −5.5, ML −1.9, DV −7.0) stereotaxically over the SN (2.5 μL /site). Injection rate was 0.5 μL/min and the needle was left in place for 10 min before it was slowly retracted from the brain.

### Behavioral analysis

The animals were tested for apomorphine-induced (0.2 mg/kg, i.p.) turning 1, 2 and 3 weeks after intracerebral injection. Only the rats that exhibited a mean rotation toward the healthy side at least 6.0 full body turns per min were considered as disease onset.

### Statistical analysis

All experiments were performed in triplicate. Data were presented as the mean ± SEM; statistical significance of difference (*P* value) was assessed using Student’s *t*-test by GraphPad Prism 5 software (GraphPad, San Diego, CA, USA), and *P* < 0.05 was considered significant.

## Electronic supplementary material

Supplementary material 1 (PDF 225 kb)
