# Supplementary material for: Secreted miR-34a in astrocytic shedding vesicles enhanced the vulnerability of dopaminergic neurons to neurotoxins by targeting Bcl-2
Source: Protein Cell. 2015 Jun 20;6(7):529–40. doi: 10.1007/s13238-015-0168-y (PMC4491052; doi:10.1007/s13238-015-0168-y)
Supplement: Supplementary file 1 — Supplementary material 1 (PDF 225 kb) [file 13238_2015_168_MOESM1_ESM.pdf]

**Figure S1**

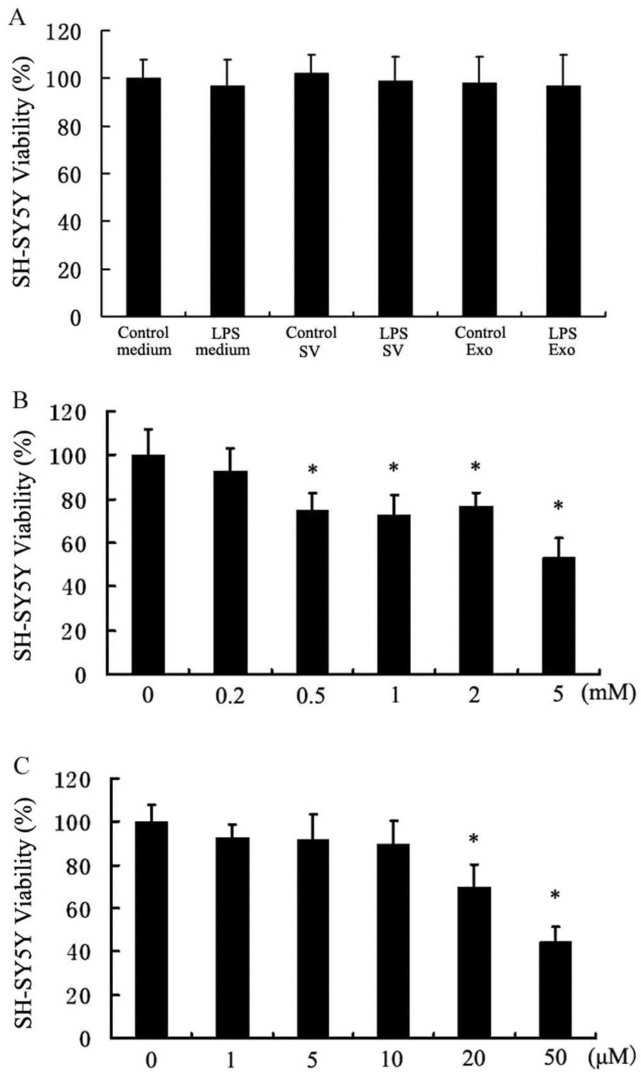


**Figure S1 Assessment of cell viability in SH-SY5Y cells with different treatments.** SH-SY5Y cell viability assessed by cck-8 assay after treatment **(A)** with conditioned medium or different types of microvesicles; **(B)** with different concentrations of MPP+, * $p<0.05$; **(C)** with different concentrations of 6-OHDA, * $p<0.05$.

**Figure S2**

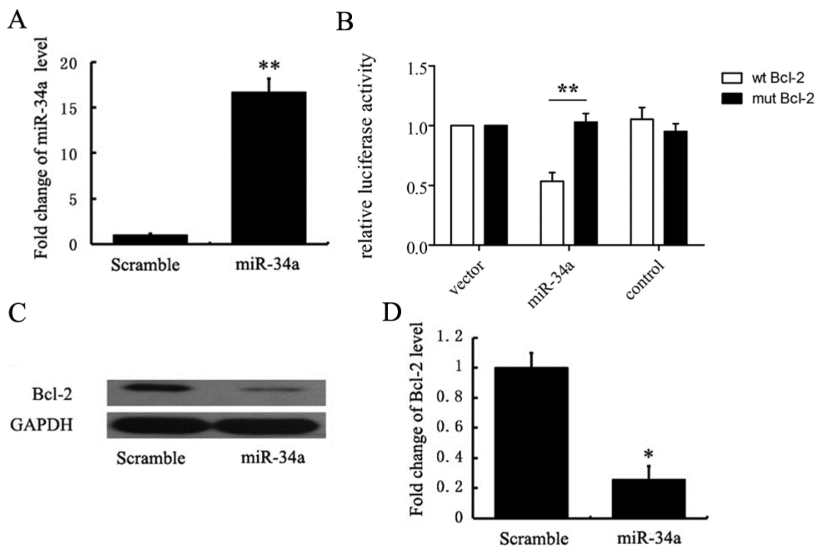


**Figure S2 Overexpression of miR-34a in SH-SY5Y cells down-regulates Bcl-2 expression level.**

**(A)** QPCR analysis showed that the level of miR-34a was significantly increased in SH-SY5Y cells after transfection, ** $p<0.01$; **(B)** Luciferase activity was measured 24 hours after transfecting SH-SY5Y cells. Reporter plasmids with the wild type (wt) or mutated (mut) 3' UTR of Bcl-2 were transfected either alone (vector) or with miR-34a mimics (miR-34a) or scrambled control (control), ** $p<0.01$; **(C)**Western-blot showed that the level of miR-34a-targeted protein Bcl-2 was decreased in SH-SY5Y cells overexpressing miR-34a; **(D)** Quantification of Bcl-2 levels in SH-SY5Y cells overexpressing miR-34a as compared with control cells, * $p<0.05$.

**Figure S3**

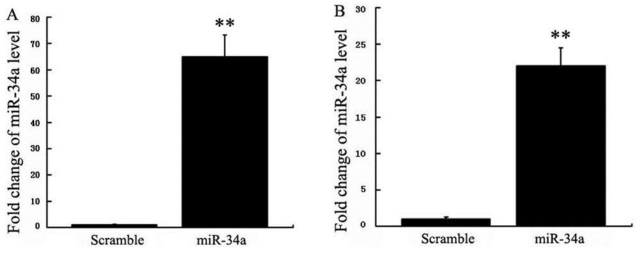


**Figure S3 Overexpression of miR-34a in U-87 MG cells leads to the increase of miR-34a level in SVs released by cells.**

**(A)** QPCR analysis showed that the level of miR-34a was increased in U-87 MG cells after transfection of miR-34a,** $p<0.01$; **(B)** QPCR analysis showed that the level of miR-34a was also significantly increased in U-87 MG cell derived SVs after transfection of miR-34a into U-87 MG cells, ** $p<0.01$.

**Figure S4**

A

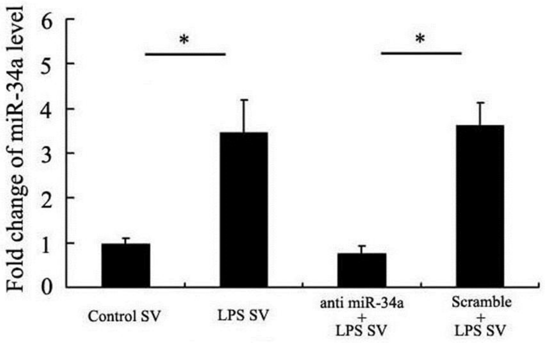


**Figure S4 QPCR analysis of miR-34a levels in different groups of SVs derived from primary astrocytes, * p<0.05.**

**Table S1 Microarray analysis of miRNA expression in control SV and LPS SV**

| **No.** | miRNA | Control SV | LPS SV | **No.** | miRNA | Control SV | LPS SV |
|---|---|---|---|---|---|---|---|
| **1** | miR-34a | 1 | 2.86 | **11** | miR-708-3p | 1 | 0.45 |
| **2** | miR-92a | 1 | 2.20 | **12** | miR-377 | 1 | 0.41 |
| **3** | miR-296-3p | 1 | 2.56 | **13** | miR-196b-3p | 1 | 0.51 |
| **4** | miR-18a | 1 | 2.47 | **14** | miR-143-5p | 1 | 0.50 |
| **5** | let-7d-3p | 1 | 2.24 | **15** | miR-455-5p | 1 | 0.41 |
| **6** | miR-214 | 1 | 2.44 | **16** | miR-188-3p | 1 | 0.45 |
| **7** | miR-216a | 1 | 2.79 | **17** | miR-761 | 1 | 0.45 |
| **8** | miR-200c-5p | 1 | 2.00 | **18** | miR-3074-3p | 1 | 0.29 |
| **9** | miR-19a | 1 | 2.23 | **19** | miR-33a | 1 | 0.29 |
| **10** | miR-194-3p | 1 | 3.02 | **20** | miR-653 | 1 | 0.30 |
